# Supplementary material for: Nuthatches vary their alarm calls based upon the source of the eavesdropped signals
Source: Nat Commun. 2020 Jan 27;11:526. doi: 10.1038/s41467-020-14414-w (PMC6985140; doi:10.1038/s41467-020-14414-w)
Supplement: Supplementary file 1 — Supplementary Information [file 41467_2020_14414_MOESM1_ESM.pdf]

Nuthatches vary their alarm calls based upon the source of the eavesdropped signals

Carlson et al.

This PDF file includes:

Supplementary Table 1

Supplementary Table 1 | Model summary. Type or Model results summary for a) call rate (calls/individual/minute), b) peak frequency (kHz), and c) call length (seconds)

a) Call Rate (call/individual/minute)

**Random effects**	**n**	**Variance**	**Std.Dev.**
Location	57	266.38	16.32
Year	5	64.59	8.04
Residual	129	2993.55	54.71

Number of obs: 129, groups: Location, 57; Year, 5

**Fixed effects**	**Estimate**	**Std. error**	***t***
Intercept	25.21	63.78	0.40
High Threat	54.47	14.90	3.66
Low Threat	48.38	16.58	2.92
Direct information	-18.73	22.26	-0.84
Order 1	-40.24	65.69	-0.61
Order 2	-38.96	43.74	-0.89
Order 3	-10.14	68.34	-0.15
Exemplar 1	138.22	84.86	1.63
Exemplar 2	42.31	46.39	0.91
Exemplar 3	1.60	83.76	0.02
Number of nuthatches	-4.35	4.00	-1.09
High Threat : Direct Information	63.75	26.28	2.43
Order 1 : Exemplar 1	-94.54	89.03	-1.06
Order 2 : Exemplar 1	-91.60	74.25	-1.23
Order 3 : Exemplar 1	-123.57	92.23	-1.34
Order 1 : Exemplar 2	-18.07	53.64	-0.34
Order 3 : Exemplar 2	-47.08	57.37	-0.82
Order 1 : Exemplar 3	11.62	88.34	0.13
Order 2 : Exemplar 3	13.52	73.02	0.19
Order 3 : Exemplar 3	1.92	91.03	0.02

b) Peak Frequency (Hz)

**Random effects**	**n**	**Variance**	**Std.Dev.**
Location	50	27627	166.2
Year	5	0	0.0
Residual	88	122440	349.9

Number of obs: 88, groups: Location, 50; Year, 5

**Fixed effects**	**Estimate**	**Std. error**	***t***
Intercept	2930.86	621.29	4.72
High Threat	179.38	131.71	1.36
Low Threat	154.82	141.59	1.09
Direct information	-310.73	161.17	-1.93
Order 1	-332.63	624.02	-0.53
Order 2	-309.34	409.43	-0.76
Order 3	-344.63	642.82	-0.54
Exemplar 1	-422.44	709.00	-0.60
Exemplar 2	-21.59	446.50	-0.05
Exemplar 3	-283.41	714.81	-0.40
Number of nuthatches	30.75	31.14	0.99
High Threat : Direct Information	652.24	199.67	3.27
Order 1 : Exemplar 1	585.01	745.42	0.79
Order 2 : Exemplar 1	674.77	578.51	1.17
Order 3 : Exemplar 1	600.29	774.64	0.78
Order 1 : Exemplar 2	187.10	514.82	0.36
Order 3 : Exemplar 2	-14.51	535.29	-0.03
Order 1 : Exemplar 3	410.60	759.46	0.54
Order 2 : Exemplar 3	326.66	597.32	0.55
Order 3 : Exemplar 3	197.33	769.06	0.26

c) Call Length (seconds)

**Random effects**	**n**	**Variance**	**Std.Dev.**
Location	50	0.004	0.061
Year	5	0.001	0.038
Residual	88	0.003	0.059

Number of obs: 88, groups: Location, 50; Year, 5

**Fixed effects**	**Estimate**	**Std. error**	***t***
Intercept	0.119	0.123	0.97
High Threat	-0.060	0.027	-2.18
Low Threat	-0.042	0.028	-1.48
Direct information	0.018	0.040	0.45
Order 1	0.053	0.124	0.43
Order 2	0.028	0.080	0.35
Order 3	0.044	0.127	0.35
Exemplar 1	0.077	0.134	0.58
Exemplar 2	0.014	0.085	0.16
Exemplar 3	-0.168	0.138	-1.22
Number of nuthatches	0.003	0.007	0.35
High Threat : Direct Information	-0.071	0.039	-1.85
Order 1 : Exemplar 1	-0.096	0.144	-0.67
Order 2 : Exemplar 1	-0.052	0.110	-0.48
Order 3 : Exemplar 1	-0.111	0.151	-0.73
Order 1 : Exemplar 2	-0.016	0.104	-0.15
Order 3 : Exemplar 2	-0.008	0.105	-0.08
Order 1 : Exemplar 3	0.126	0.147	0.86
Order 2 : Exemplar 3	0.207	0.117	1.78
Order 3 : Exemplar 3	0.182	0.148	1.23
