# Supplementary material for: Nuthatches vary their alarm calls based upon the source of the eavesdropped signals
Source: Nat Commun. 2020 Jan 27;11:526. doi: 10.1038/s41467-020-14414-w (PMC6985140; doi:10.1038/s41467-020-14414-w)
Supplement: Supplementary file 3 — Reporting Summary [file 41467_2020_14414_MOESM3_ESM.pdf]

nature research

Corresponding author(s): Nora V Carlson

Last updated by author(s): Dec 14, 2019

Reporting Summary

Nature Research wishes to improve the reproducibility of the work that we publish. This form provides structure for consistency and transparency in reporting. For further information on Nature Research policies, seeAuthors & Referees and theEditorial Policy Checklist .

Statistics

For all statistical analyses, confirm that the following items are present in the figure legend, table legend, main text, or Methods section.

n/a	Confirmed	
☐	☒	The exact sample size (*n*) for each experimental group/condition, given as a discrete number and unit of measurement
☐	☒	A statement on whether measurements were taken from distinct samples or whether the same sample was measured repeatedly
☐	☒	The statistical test(s) used AND whether they are one- or two-sided<br>*Only common tests should be described solely by name; describe more complex techniques in the Methods section.*
☐	☒	A description of all covariates tested
☐	☒	A description of any assumptions or corrections, such as tests of normality and adjustment for multiple comparisons
☐	☒	A full description of the statistical parameters including central tendency (e.g. means) or other basic estimates (e.g. regression coefficient) AND variation (e.g. standard deviation) or associated estimates of uncertainty (e.g. confidence intervals)
☐	☒	For null hypothesis testing, the test statistic (e.g. *F*, *t*, *r*) with confidence intervals, effect sizes, degrees of freedom and *P* value noted<br>*Give P values as exact values whenever suitable.*
☒	☐	For Bayesian analysis, information on the choice of priors and Markov chain Monte Carlo settings
☒	☐	For hierarchical and complex designs, identification of the appropriate level for tests and full reporting of outcomes
☒	☐	Estimates of effect sizes (e.g. Cohen's *d*, Pearson's *r*), indicating how they were calculated

Our web collection on statistics for biologists contains articles on many of the points above.

Software and code

Policy information about availability of computer code

Data collection	We did not use any code to collect any of the data in this study.
Data analysis	We used Raven Pro acoustical software version 1.4 and 1.5, as well as R version 3.6.1

For manuscripts utilizing custom algorithms or software that are central to the research but not yet described in published literature, software must be made available to editors/reviewers. We strongly encourage code deposition in a community repository (e.g. GitHub). See the Nature Research guidelines for submitting code & software for further information.

Data

Policy information about availability of data

All manuscripts must include a data availability statement. This statement should provide the following information, where applicable:

- Accession codes, unique identifiers, or web links for publicly available datasets
- A list of figures that have associated raw data
- A description of any restrictions on data availability

Data and R Code used in this article for analysis and Figure 2 can be found in Source Data 1 and Supplementary Code 1 respectively.

Field-specific reporting

Please select the one below that is the best fit for your research. If you are not sure, read the appropriate sections before making your selection.

☐ Life sciences ☐ Behavioural & social sciences ☒ Ecological, evolutionary & environmental sciences

For a reference copy of the document with all sections, see nature.com/documents/nr-reporting-summary-flat.pdf

Ecological, evolutionary & environmental sciences study design

All studies must disclose on these points even when the disclosure is negative.

Study description	We conducted a repeated measures playback experiment on a population of red-breasted nuthatches in the wild over a number of years. For this study we examined how three acoustic measures of nuthatch mob calls (call rate, peak frequency, and call length) varied in response to information about predator threat from both direct (predator calls) and indirect (heterospecific mobbing calls) sources. To analyze this data we used linear mixed models with a Gaussian distribution. While we had a repeated measures design (intended for every individual to receive all 5 playback treatments) due to variation in sampling effort, sample sizes varied somewhat across treatments: control (n=38), low-threat direct-information (n=19), low-threat indirect-information (n=29), high-threat indirect-information (n=35), and high-threat direct-information (n=21).
Research sample	We chose red-breasted nuthatches as they fit a number of criteria needed for this study (tend to stay on year-round territories, but readily join mixed-species flocks with chickadees and other species, eavesdrop and respond correctly to chickadee mobbing calls, and produce mobbing calls of their own), and we wanted to build off a previous study (Templeton et al. 2007). As we wanted to build on the work conducted in a previous study, we decided to use the same population for the majority of the experiments. For a small portion of the experiments we decided to use a similar but different population due to logistics, and availability. As the second population lives in a similar habitat with the same species composition, they were equivalent populations for this study.
Sampling strategy	We did not predetermine the number of samples needed, but as we had troubles collecting a sample size we were happy with a number of years in a row, due to a number of factors, including years it was difficult to locate nuthatches, and a lack of funding for this project in later years, we continued to collect samples until we deemed our sample size reasonable.
Data collection	All authors collected data in the following manner: A nuthatch not currently flocking in a mixed species flock with chickadees was located by sight or sound at a feeder site. The experimenter placed a field speaker at the base of a tree near the feeder and began the playback. The playback lasted for one minute. During and after the playback, the experimenter (from a minimum of 4 meters away) would record all of the vocalizations produced by the nuthatch. After about 10 minutes the experimenter would retrieve the speaker and leave the site.
Timing and spatial scale	Playbacks were conducted from: 27 November 2006 – 6 December 2007, 1 March - 29 March 2013, 17 November 2014 – 17 December 2015, 27 December – 29 December 2016, and 23 February – 2 March 2017. The data were taken from in and around Missoula Montana and Mazama Washington with no two sites closer than 500 meters.
Data exclusions	Occasionally nuthatches would respond to live raptors that flew by just before or during the experiments; we excluded these experiments form the analyses.
Reproducibility	While we conducted these experiments over a number of years, we have not reproduced this experiment since.
Randomization	For each focal groups of nuthatches, the order of presentation of the stimuli and the exemplar for each stimulus were randomized.
Blinding	As both the playback presentation and the processing of the recordings with playbacks recorded on them included the playback treatments, the experimenters conducting the playbacks or processing the recordings could not be blind to the treatment.

Did the study involve field work? ☒ Yes ☐ No

Field work, collection and transport

Field conditions	Playbacks were conducted during the winters of 2005 17, and 2012, 2013, and 2015, and 2016 and the weather was typically below freezing and snowy.
Location	All experiments took place in and around Missoula, Montana (46°, 50′ N; 114°, 02′ W), and in and around Mazama, Washington (48°, 35′ N; 120°, 24′ W).
Access and import/export	Research was conducted under the University of Montana's Institutional Animal Care and Use Committee's Animal Use Protocols (ACC 022-01, 049-14EGDBS- 080814, 001-11EGDBS-080511). All sites were on private property with owner's permission at bird feeders. They were all in easily accessible areas, limiting the effect of our effect on the environment when accessing the feeders.
Disturbance	Non-control playbacks induced mobbing behaviour in many cases which disturbed the birds' normal foraging behaviour, however they generally quickly returned to normal foraging within 10 minutes of the end of a playback. Additionally, we conducted our experiments to periods between one hour after sunrise and one hour before sunset to allow birds to feed after waking up and before the night.

Reporting for specific materials, systems and methods

We require information from authors about some types of materials, experimental systems and methods used in many studies. Here, indicate whether each material, system or method listed is relevant to your study. If you are not sure if a list item applies to your research, read the appropriate section before selecting a response.

Materials & experimental systems

n/a	Involved in the study
☒	☐ Antibodies
☒	☐ Eukaryotic cell lines
☒	☐ Palaeontology
☐	☒ Animals and other organisms
☒	☐ Human research participants
☒	☐ Clinical data

Methods

n/a	Involved in the study
☒	☐ ChIP-seq
☒	☐ Flow cytometry
☒	☐ MRI-based neuroimaging

Animals and other organisms

Policy information about studies involving animals; ARRIVE guidelines recommended for reporting animal research

Laboratory animals	We only used wild animals.
Wild animals	We conducted auditory playback to pairs of nuthatches at 60 locations in and around Missoula, Montana (46°, 50' N; 114°, 02' W) during the winters of 2005 17, and 2012, 2013, and 2015, and in three locations in and around Mazama, Washington (48°, 35' N; 120°, 24' W) during the winter of 2016. We used a repeated measures experimental design where individuals at each location were presented with each of the five playback stimuli (high-threat northern pygmy-owl call, low threat great-horned owl call, high threat chickadee mobbing call, low threat chickadee mobbing call, and Townsend's solitaire or house sparrow song control; with the exception of the 2007 data used from 17, in which birds only received chickadee call and control playbacks). We could not be sure of individual identity, sex, or age for all trials as individuals were not colour ringed. Playbacks lasted one minute, with a natural peak amplitude of 75 dB SPL (A-weighting), and were started when a nuthatch not currently affiliated with a mixed-species group was located by ear or eye. We conducted our experiments to periods between one hour after sunrise and one hour before sunset to allow birds to feed after waking up and before the night.
Field-collected samples	We only collected acoustic samples (i.e. audio recordings of their vocal response to playback experiments).
Ethics oversight	Our experiments conformed to the standards outlined in the ASAB/ABS Guidelines for the Use of Animals in Research and were conducted under the University of Montana's Institutional Animal Care and Use Committee's Animal Use Protocols (ACC 022-01, 049-14EGDBS- 080814, 001-11EGDBS-080511).

Note that full information on the approval of the study protocol must also be provided in the manuscript.
