# Supplementary material for: Nuthatches vary their alarm calls based upon the source of the eavesdropped signals
Source: Nat Commun. 2020 Jan 27;11:526. doi: 10.1038/s41467-020-14414-w (PMC6985140; doi:10.1038/s41467-020-14414-w)
Supplement: Supplementary file 4 — Description of Additional Supplementary Files [file 41467_2020_14414_MOESM4_ESM.pdf]

Description of Additional Supplementary Files

File Name: Supplementary Data 1
Description: **Data used for all analyses.** This is the data analyzed for this paper. Each row is one trial at a location. It includes the following columns: Location (the location the trial was at), Stimulus (the playback stimulus used), Threat (the threat level of the stimulus used), Information (the source (direct vs. indirect) of the stimulus), Exemplar (the number of the playback used), Day (the day on which the experiment was conducted), Month (the month during which the experiment was conducted), Year (the year during which the experiment was conducted), Order (the order of the presentation), RBNU.num (the number of nuthatches present), raw.calls (the total number of nuthatch calls produced), call.rate (the number of calls divided by the number of nuthatches present and by the time), pfreq (the average peak frequency of the calls in Hz), length (the average call length in seconds).

File Name: Supplementary Movie 1
Description: **Rotating 3D graph of all acoustic features plotted against one another.** A 3D rotating graph showing each of the acoustic parameters -- peak frequency (kHz), call rate (calls/min), and call length (seconds) -- graphed against one another to better visualize the relationship between variables and the separation between the control (grey triangle) direct (blue circle) and indirect (orange square) information.

File Name: Supplementary Software 1
Description: **R code used in all analyses.** This code was used to analyze the data. It includes the three libraries needed (lme4, car, and readxl), loading the data (Supplementary Data 1.xslx), and the code used to run linear mixed models on the data (LMM call rate, LMM peak frequency, LMM call length).
